# Supplementary material for: Antiarrhythmic Effect of Sacubitril-Valsartan: Cause or Consequence of Clinical Improvement?
Source: J Clin Med. 2019 Jun 18;8(6):869. doi: 10.3390/jcm8060869 (PMC6616876; doi:10.3390/jcm8060869)
Supplement: Supplementary file 1 [file jcm-08-00869-s001.pdf]

Table S1. **Baseline characteristics**

Characteristics
Age
Etiology
Gender
NYHA class
Body mass index
Heart failure hospitalization in the previous year
Smokers
Previous hypertension
Dyslipidemia
Diabetes mellitus
Peripheral artery disease
Familiar history of Heart Failure
Atrial Fibrillation
Chronic kidney disease
Chronic liver disease
Angiotensin-converting enzyme inhibitors
Angiotensin II receptor blocker
Beta-blockers
Mineralocorticoid receptor antagonist
Ivabradine
Digoxin
Implantable cardioverter defibrillator
Cardiac resynchronization therapy
Percutaneous mitral-valve repair using a MitraClip®

Table S2. **Outcomes**

Outcomes
Death
Cardiac death
Sudden cardiac death
Non-cardiac death
Heart failure hospitalization
Need for inotropic support
Heart transplantation
Left ventricular assist device
Adverse events requiring discontinuation

Table S3. **Heart Failure Prognostic Scores**

Heart Failure Prognostic Scores
Heart Failure Survival Score
Seattle Heart Failure Model (1 year expected survival)
Seattle Heart Failure Model (5 years expected survival)
MAGGIC score
1 year MAGGIC score risk of dying
3 years MAGGIC score risk of dying

Table S4. Laboratorial Data

Laboratorial Data
Hemoglobin (mg/dL)
Mean corpuscular volume (fL)
Ferritin (ng/mL)
INR
Total bilirubin (mg/dL)
Aspartate transaminase (U/L)
Alanine transaminase (U/L)
Glomerular filtration rate (mL/min)
Creatinine (mg/dL)
Urea (mg/dL)
Potassium (mEq/L)
Sodium (mEq/L)
Creatine Kinase (U/L)
Uric acid (mg/dL)
Glycated hemoglobin (%)
Thyroid-stimulating hormone (mU/L)
Creatine Kinase (U/L)
Troponin I (pg/mL)
NT-proBNP (pg/mL)
BNP (pg/mL)

Table S5. Electrocardiographic Data

Electrocardiographic Data
Heart rate (bpm)
PQ interval (ms)
QRS interval (ms)
QTc interval (ms)
SV2 + RV5 (mm)
Biventricular pacing (%)
Cardiac rhythm
Intraventricular conduction block
Presence of Q waves

Table S6. Cardiopulmonary Exercise Test Data

Cardiopulmonary Exercise Test Data
Maximal heart rate (bpm)
Maximal predicted heart rate (%)
First minute heart rate recovery (bpm)
Initial systolic blood pressure (mmHg)
Maximal systolic blood pressure (mmHg)
Peak oxygen consumption (mL/kg/min)
Peak predicted oxygen consumption (%)
VE/VCO_2 slope
Peak ratio exchange ratio
Duration of exercise (s)
Duration of exercise until anaerobic threshold (s)
Oxygen consumption at anaerobic threshold (mL/kg/min)

Table S7. **Echocardiographic Data**

**Echocardiographic Data**
Left ventricle end-diastolic diameter (mm)
Left ventricle end-systolic diameter (mm)
Interventricular septum (mm)
Left ventricular ejection fraction (%)
Global longitudinal strain (%)
Myocardial Constructive Work (mmHg)
Myocardial Wasted Work (mmHg)
Myocardial work index (mmHg%)
Myocardial Work Efficiency (%)
Mechanical dispersion index
E/e´
Pulmonary artery systolic pressure (mmHg)
Left atrium volume (mL/m^2)
Right atrium volume (mL/m^2)
Mechanical dispersion (ms)
Diastolic dysfunction grade
Mitral regurgitation grade
Tricuspid regurgitation grade
Aortic regurgitation grade
E (cm/s)
A (cm/s)
Mean e´ (cm/s)
Tricuspid regurgitant velocity (m/s)
Presence of pericardial effusion
Inferior vena cava index
